# Vaccine effectiveness against the B.1.617.2 in the intensive care unit

**DOI:** 10.1097/MD.0000000000035588

**Published:** 2023-10-20

**Authors:** Ibrahim Koc, Sevda Unalli Ozmen, Olgun Deniz

**Affiliations:** a Bursa City Hospital Pulmonary Medicine, Bursa, Turkey; b Bursa City Hospital Medical Biochemistry, Bursa, Turkey; c Bursa City Hospital, Palliative Care Unit, Geriatric Medicine Clinic, Bursa, Turkey.

**Keywords:** COVID-19, survival, the B.1.617.2, vaccine

## Abstract

Severe acute respiratory syndrome-coronavirus 2 and its variants are still a concern for the World. The effectiveness of the BioNTech and Sinovac vaccines against the B.1.617.2 variant, particularly in the intensive care unit, has been unclear. This study aimed to investigate the vaccine effectiveness of BioNTech and Sinovac vaccines in reducing severe disease, intubation, and mortality rates in B.1.617.2 infected patients followed in the intensive care unit. The data of 208 unvaccinated and 234 vaccinated B.1.617.2 variants were retrospectively reviewed. Severe disease status, complaints, the percent oxygen saturation in the blood at the first admission, and other clinical information during follow-up were recorded. With the BioNTech and Sinovac vaccines being the most common in the region, mortality rate, severe disease, and intubation were more frequent in the unvaccinated group. As for survival rates, 58.5 (137) of the vaccinated and 35.1 % (73) of the unvaccinated survived. In the vaccinated group, 64.3 % (27) of vaccinated with 3 Sinovac, 80 % (16) of 2 Sinovac and 1 BioNTech, and 71.7 % of 2 BioNTech survived. Vaccination with 2 doses of BioNTech and 3 doses of Sinovac reduces mortality. Furthermore, 2 doses of Sinovac and 1 dose of BioNTech are more protective.

## 1. Introduction

At the end of 2019, the world met with the coronavirus disease-2019 (COVID-19) disease, which will upset all balances and cause the death of millions of people and the illness of many more in the following period. The disease caused by the severe acute respiratory syndrome-coronavirus 2 (SARS-CoV-2) virus is highly transmissible and has spread worldwide in the following times. At the beginning of the pandemic, the world seemed helpless. The SARS-CoV-2 has proven to be more contagious than other types of coronaviruses, even in people with no symptoms.^[1]^ The effects of COVID-19 are not only in the health field, but the lockdown disrupts the supply-demand balance and harms the global economy. Patient overload in COVID-19 clinics and intensive care units caused health systems to collapse. The scientific world was desperately looking for a cure at the pandemic’s beginning. Treatments including azithromycin and hydroxychloroquine have been proven successful.^[2]^ However, there were no significant results in other studies.^[3,4]^ Proper use of masks, social distancing, and hygiene are still substantially placed in reducing transmission. However, medical and conservative treatment and vaccination are vital to control the disease in ongoing life. Therefore, the search for an effective vaccine against SARS-CoV-2 has been the main topic of research to help restabilize the average pace. In late March 2021, India experienced a surge in cases of COVID-19, causing thousands of deaths.^[5]^ The B.1.617.2 variant has now been detected across the globe. The effectiveness of the Pfizer–BioNTech (BNT162b2) and Sinovac vaccines against this variant has been unclear, and there is less data about their efficacy in the intensive care unit (ICU). The present study aimed to investigate the vaccine effectiveness (VE) of BioNTech and Sinovac vaccines in reducing severe disease, intubation, and mortality rates in those patients infected with B.1.617.2 followed in the ICU. At the time of the study, both vaccines were available, and one of the 2 vaccines was administered. The second dose was given 1 month after the first dose, and the following doses at 3-month intervals, according to the patient’s preference, after the physician’s explanation.

## 2. Materials and methods

The data of patients infected with B.1.617.2 and hospitalized in ICU in a tertiary hospital/ in Turkey between May 2021 and December 2021 were retrospectively investigated. Out of them, 208 unvaccinated and 234 vaccinated B.1.617.2 variants were included in the study. Vaccination-related information was obtained from the hospital database. The BioNTech and Sinovac vaccines are more common in the region. The ethics committee approval was obtained from a tertiary city hospital Clinical Research Ethics Committee in Turkey. Vaccination status, gender of the patients, age distribution, comorbidities, length of stay in a clinical and intensive care unit (ICU), severe disease status, intubation, and mortality rates were recorded for each patient. Severe disease status, complaints, and the percent oxygen saturation in the blood (SpO_2_) were recorded at the first admission, and other clinical information during their follow-up. Statistical package for the social sciences (SPSS) version 25.0 was used for statistical analyses. Normally distributed parameters were compared by the Student *t* test and others by the Mann–Whitney *U* test. Categorical variables were compared by Chi-square or Fisher Exact tests, where appropriate. Categorical variables were presented as number and frequency, and a *P* value < .05 was considered statistically significant. Multivariate binary logistic regression was used to identify independent predictors associated with in-hospital mortality. Hosmer–Lemeshow goodness of fit statistics was performed to assess model fit. Variables that remained significant (*P* < .05) in the multivariate model were considered independent predictors for in-hospital mortality.

## 3. Results

Four hundred and forty-two patients infected with the B.1.617.2 variants and followed in the ICU were included in the study. The mean age was determined to be 68 ± 12.7 in the unvaccinated group and 70 ± 12.6 in the vaccinated group (Table 1). Out of 442 patients, 208 were unvaccinated, and 234 were vaccinated with at least 1 dose of BioNTceh or Sinovac. In the vaccinated group, the rate of patients immunized with a single dose of Sinovac was 12 (5.1%), 2 doses of Sinovac 90 (38.5%), 3 doses of Sinovac 41 (17.5%), 2 Sinovac and 1 BioNTech 20 (8.5%), a single dose of BioNTech 11 (4.7%) and 2 doses of BioNTech 60 (25.6%). In the vaccinated group, cough, joint pain, and fever were more common complaints, whereas mortality rate, severe disease, and intubation were more frequent in the unvaccinated group (Table 1). Regarding in-hospital mortality, 41.5 % (97) of vaccinated patients (at least 1 dose of BioNTech or Sinovac) died, and 58.5% (137) survived. In the unvaccinated group, 64.9% (135) of patients died. Among all patients vaccinated with 3 doses of Sinovac, 64.3 % (27), 2 Sinovac and 1 BioNTech 80% (16), and 71.7 % of patients vaccinated with 2 doses of BioNTech survived. Sixty-nine point five% of intubated patients (141) and 62.6 % (209) of patients admitted with severe disease died (Table 2). The variables identified to be significantly associated via univariate analysis were included in the binary logistic regression analysis to detect the possible ICU mortality parameters. Multivariate analysis revealed that patients who presented at first admission with cough and severe disease and were intubated during their follow-up conferred an increased risk of mortality (Table 3). Vaccination with 2 Sinovac and 1 dose of BioNTech, 2 BioNTech, 3 Sinovac, and higher levels of SpO_2_ at first admission had a decreased risk of mortality (odds ratio [OR]:0.1, 0.24, 0.27, and 0.94, respectively).

**Table 1:** Unvaccinated and vaccinated patients infected with B.1.617.2.

Variable	Unvaccinated n = 208	Vaccinated n = 234	*P* value
Age (yr)	68 ± 12.7	70 ± 12.6	.07
Female n (%)	105 (50.5%)	98 (48.3%)	.07
Male n (%)	103 (49.5%)	136 (58.1%)	
Single dose of Sinovac, n (%)	-	12 (5.1%)	
Two Sinovac, n (%)	-	90 (38.5%)	
Three Sinovac, n (%)	-	41 (17.5%)	
Two Sinovac and one BioNTech, n (%)	-	20 (8.5%)	
Single dose of BioNTech, n (%)	-	11 (4.7%)	
Two BioNTech, n (%)	-	60 (25.6%)	
Shortness of breath	79 (38%)	83 (35.5%)	.5
Cough n (%)	48 (23.1)	116 (49.6%)	.001*
Fatigue n (%)	45 (21.6%)	41 (17.5%)	.27
Joint pain n (%)	34 (16.3%)	83 (35.5%)	.001*
Fever n (%)	31 (14.9%)	58 (24.8%)	.01*
GIS symptoms n (%)	41 (19.7%)	36 815.4%)	.2
Mortality, n (%)	135 (64.9%)	97 (41.5%)	.001*
Severe disease n (%)	177 (85.1%)	157 (67.1%)	.001*
Clinic stay (d)	3 (0–23)	2 (0–20)	.04*
ICU stay (d)	11 (2–25)	10 (2–21)	.02*
Intubation n (%)	113 (54.3%)	90 (38.5%)	.001*
SpO_2_ %	87 (60–99)	89 (65–97)	.01*
COPD n (%)	15 (7.2%)	36 (15.4%)	.007*
Asthma, n (%)	7 (3.4%)	28 (12%)	.001*
Diabetes mellitus, n (%)	57 (27.4%)	74 (31.6%)	.3
Hypertension, n (%)	93 (44.7%)	124 (53%)	.08
Coronary artery disease, n (%)	36 (17.3%)	70 (29.9%)	.002*
Malignity, n (%)	12 (5.8%)	30 (12.8%)	.01*
Chronic kidney disease, n (%)	3 (1.4%)	14 (6%)	.01*

COPD = chronic obstructive pulmonary disease, GIS = gastrointestinal system, ICU = intensive care unit, SpO_2_ = the percent oxygen saturation in the blood.

**Table 2:** The results of the study regarding in-hospital mortality.

Variable	In-hospital mortality		*P* value
	No (n = 210)	Yes (n = 232)	
Age (yr)	66.7 ± 11	70 ± 11.2	.006*
Female n (%)	99 (48.8%)	104 (51.2%)	.6
Male n (%)	111 (46.4%)	128 (53.6%)	
Vaccinated n (%)	137 (58.5%)	97 (41.5%)	.001*
Unvaccinated n (%)	73 (35.1%)	135 (64.9%)	.001*
Single dose of Sinovac, n (%)	5 (41.7%)	7 (58.3%)	.6
Two Sinovac, n (%)	41 (45.6%)	49 (54.4%)	.67
Three Sinovac, n (%)	27 (64.3%)	15 (35.7%)	.02*
Two Sinovac and one BioNTech, n (%)	16 (80%)	4 (20%)	.003*
Single dose of BioNTech, n (%)	6 (54.5%)	5 (45.5%)	.6
Two BioNTech, n (%)	43 (71.7%)	17 (28.3%)	.001*
Shortness of breath n (%)	87 (53.7%)	75 (46.3%)	.04*
Cough n (%)	79 (48.2%)	85 (51.8%)	.8
Tiredness n (%)	42 (48.8%)	44 (51.2%)	.7
Joint pain n (%)	57 (48.7%)	60 (51.3%)	.7
Fever n (%)	43 (48.3%)	46 (51.7%)	.8
GIS symptoms n (%)	39 (50.6%)	38 (49.4%)	.5
Severe disease n (%)	125 (37.4%)	209 (62.6%)	.001*
Clinic stay n (%)	3 (0-23)	2 (0-20)	.01*
ICU stay n (%)	10 (2-24)	11 (2-25)	.03*
Inbutaed n (%)	62 (30.5%)	141 (69.5%)	.001*
SpO2 %	90 (65-97)	85 (60-99)	.001*
Chonic obtructive pulmonary disease n (%)	21 (41.2%)	30 (58.8%)	.08
Asthma, n (%)	17 (48.6%)	18 (51.4%)	.89
Diabetes Mellitus, n (%)	62 (47.3%)	69 (52.7%)	.9
Hypertension, n (%)	105 (48.4%)	112 (51.6%)	.7
Coronary artery disease, n (%)	52 (49.1%)	54 (50.9%)	.7
Malignity, n (%)	29 (69%)	13 (31%)	.003*
Chronic kidney disease, n (%)	10 (58.8%)	7 841.2%)	.34

GIS = gastrointestinal system, ICU = intensive care unit, SpO_2_ = the percent oxygen saturation in the blood.

**Table 3:** Binary logistic regression to determine the mortality risk.

Variables	OR	*P* value
Two Sinovac and one BioNTech	0.1 (0.025–0.4)	.001*
Two BioNTech	0.24 (0.11–0.5)	.001*
Three Sinovac	0.27 (0.11–0.64)	.003*
Cough	2.06 (1.23–3.4)	.006*
Severe disease	4 (2.2–7.2)	.001*
SpO_2_	0.94 (0.91–0.97)	.001*
Intubation	3.2 (2.02–5.2)	.001*

Data in parentheses are at a 95% confidence interval.

OR = odds ratio, SpO_2_ = the percent saturation of oxygen in the blood.

* *P* < .05 indicates statistical significance.

## 4. Discussion

Since COVID-19 broke out, the scientific world has started to look for treatment methods. Besides the effective antiviral medications for COVID-19 treatment, strengthening the immune system reduces the risk of illness, and helps patients get over the condition with minor damage. Previously it was reported that the messenger RNA vaccine BNT162b2 had 95% efficacy against COVID-19.^[6]^ Other reports confirmed effectiveness among healthcare workers.^[7]^ Even though vaccination reduces the risk of disease, infections can still occur after or even during the completion of the vaccination schedule, which is so-called breakthrough cases. In a report from Qatar, VE against severe, critical, or fatal disease due to infection with any SARS-CoV-2 (with the B.1.1.7 and B.1.351 variants predominant at the time the study was conducted in Qatar) was found to be 97.4%.^[8]^ In the present study, patients who received at least 1 dose of both vaccines had less severe disease rates. COVID-19 affects the respiratory system and many other organ systems, including the neurologic system,^[9]^ the heart^[10]^ and the gastrointestinal system.^[11]^ Chronic obstructive pulmonary disease (COPD) ranks high as a cause of death worldwide, but there is no clear evidence if it increases COVID-19 susceptibility. Patients with COPD have impaired immune responses and frequent respiratory infections. They demonstrate delayed clearance of respiratory viruses, and there are reports about poor outcomes in patients with COVID-19.^[12,13]^ However, there is not enough information about the B.1.617.2 variant. In the present study, 58 % of patients with COPD could not survive in the ICU. However, in the analyses performed, COPD was not an independent risk factor for mortality. Fever and cough were reported to be more frequent complaints among COVID-19 patients.^[14]^ In the present study, as cough was more common among vaccinated patients, this situation might be because the vaccinated group had higher rates of asthma and COPD. But we do not know if this situation is related to vaccines. Patients with COVID-19 may suffer from pains and aches, particularly joint pain. In the present study, joint pain was more common among vaccinated B.1.617.2. Even though the reason is unknown, it might be due to higher comorbidity rates such as malignancy. The percent oxygen saturation in the blood is a vital parameter indicating well-being. Previous studies reported the B.1.617.2 variant to be more mortal than other variants.^[15]^ In the present study, even though vaccinated B.1.617.2 variants had higher rates of asthma and COPD, they had higher SpO_2_ levels at admission, which was associated with a decreased risk of mortality (OR: 0.94). Else-more intubation and severe disease rates, which were associated with increased risk of mortality, were higher in the unvaccinated group.

High death rates were reported early in the pandemic due to massive cases and disruptions in the health system’s response.^[16]^ The mortality rate is higher in the patient group, primarily elderly, and has comorbidities. The B.1.617.2 variant was previously reported to cause more mortal cases than other variants.^[15,17]^ Yet little is known about VE in the ICU. Intubation and severe disease are undoubtedly the precursors of the worsening course of the disease. In this study, the rate of severe disease at the first admission and intubation during follow-up was lower in the vaccinated group. In the fight against the COVID-19 pandemic, it is essential to prevent the transmission of the disease and for patients to receive appropriate treatment. However, the first goal of patients hospitalized in the ICU is undoubtedly the survival of the patients and their discharge from the ICU. In the present study, despite a higher rate of comorbidities, 58.5 % of vaccinated patients did survive, whereas the survival rate was 35.1 % in the unvaccinated group. Else-more patients who received at least 1 dose of both vaccines had shorter clinical and intensive care unit stays.

Inactivated vaccines (CoronaVac) were reported to be safe for use previously.^[18,19]^ Duarte et al^[20]^ reported breakthrough cases were primarily mild after CoronaVac. In a study from China, Li et al^[21]^ reported VE of a single dose of inactivated SARS-CoV-2 vaccine yielded 13% efficacy while 2-dose vaccination was 100 % effective against severe COVID-19. In the present study, 58 % of patients vaccinated with a single dose and 54.4 % with 2 doses of Sinovac could not survive. The discrepancy between studies might be due to the heterogeneity of COVID-19 or the B.1.617.2 variant.

Previous studies supported the high effectiveness of BNT162b2 against hospital admissions up until around 6 months after being fully vaccinated.^[22]^ A study from Hong Kong reported that vaccination with BNT162b2 induces more robust humoral responses than CoronaVac.^[23]^ In the present study, comparing 2 doses of vaccination, 71.7 % of patients had 2 doses of BioNTech, whereas 45 % of patients vaccinated with Sinovac survived. Vaccination with 2 doses of Sinovac was an independent factor for decreasing mortality (OR: 0.24). In light of the obtained results, BioNTech seems to be more effective than Sinovac in reducing the mortality of B.1.617.2 in the ICU. Even though 2 doses of Sinovac and a single dose of BioNTech did not decrease mortality rates, 80% of patients who had 2 Sinovac and 1 dose of BioNTech did survive else-more the same vaccination status was more effective in reducing mortality than 2 doses of BioNTech and 3 doses of Sinovac.

This study has limitations; besides being a retrospective study, although the total number of cases is high, the number of cases per group decreases when divided by vaccination status. For example, in the vaccinated group, some patients had a single dose of BioNTech or Sinovac, which did not affect mortality. The intervals between the last dose of vaccine and admission to the intensive care unit could not be reached which may have affected the results.

## 5. Conclusion

A single dose of Sinovac or BioNTech does not affect survival. In contrast, vaccination with 2 doses of BioNTech and 3 doses of Sinovac reduces the mortality rate of B.1.617.2 in the ICU. Furthermore, vaccination with 2 doses of Sinovac and 1 dose of BioNTech is more protective and an independent factor in decreasing mortality.

## Author contributions

**Conceptualization:** Ibrahim Koc, Olgun Deniz.

**Data curation:** Ibrahim Koc.

**Formal analysis:** Ibrahim Koc, Sevda Unalli Ozmen, Olgun Deniz.

**Funding acquisition:** Ibrahim Koc.

**Investigation:** Ibrahim Koc.

**Methodology:** Ibrahim Koc, Sevda Unalli Ozmen, Olgun Deniz.

**Project administration:** Ibrahim Koc, Olgun Deniz.

**Resources:** Ibrahim Koc, Sevda Unalli Ozmen.

**Software:** Ibrahim Koc.

**Supervision:** Ibrahim Koc, Olgun Deniz.

**Validation:** Ibrahim Koc, Sevda Unalli Ozmen.

**Visualization:** Ibrahim Koc, Olgun Deniz.

**Writing – original draft:** Ibrahim Koc, Sevda Unalli Ozmen.

**Writing – review & editing:** Ibrahim Koc.
